# Localized Scleroderma Associated with Chronic Hepatitis C

**DOI:** 10.1155/2012/743896

**Published:** 2012-11-21

**Authors:** Felipe Ladeira de Oliveira, Luisa Kelmer Côrtes de Barros Silveira, Maria Lourdes Candela Rambaldi, Fabio Cuiabano Barbosa

**Affiliations:** General Dermatology Department, Dermatology Clinic Dr. Fabio Cuiabano Barbosa, Rua Visconde de Pirajá 330, Ipanema, 22410-000 Rio de Janeiro, RJ, Brazil

## Abstract

Hepatitis C virus has been associated with various skin conditions, such as porphyria cutanea tarda and lichen planus, as an example. The objective of this paper is based on the description of a case of localized morphea, which came years after the discovery of hepatitis C, as well as a discussion of possible relations between both diseases.

## 1. Introduction

Localized scleroderma (LS) is a disorder of the connective tissue, characterized by thickening or sclerosis of the dermis. This occurs due to the excessive deposition of collagen fibers, a fact that makes the skin hardened and rigid [1]. It is clinically classified into various forms: plaques, generalized, linear, and subtypes according to the depth of the tissue involved and its presentation [1–3]. 

The plaque morphology is the most common subtype of LS, presenting single or multiple ivory-white plaques, circumscribed, sometimes confluent, fibrotic, and reaching up to 20 cm in diameter [1, 2]. During active phases, lesions often expand presenting violet borders, called lilac rings, which correspond to the perceptible inflammatory halo seen in its histopathology exam [2, 3].

The Hepatitis C virus (HCV) has been associated with various skin conditions, such as porphyria cutanea tarda, lichen planus, mixed cryoglobulinemia, erythema nodosum, erythema multiforme, and even systemic scleroderma [4–6]. However, so far in the literature, there are few reports of the association of localized scleroderma or morphea with HCV [5, 6].

The objective of this report is based on the description of a case of morphea *en plaque*, which came years after the discovery of hepatitis C, as well as a discussion of possible relations between both diseases.

## 2. Case Presentation

**Figure 1:**
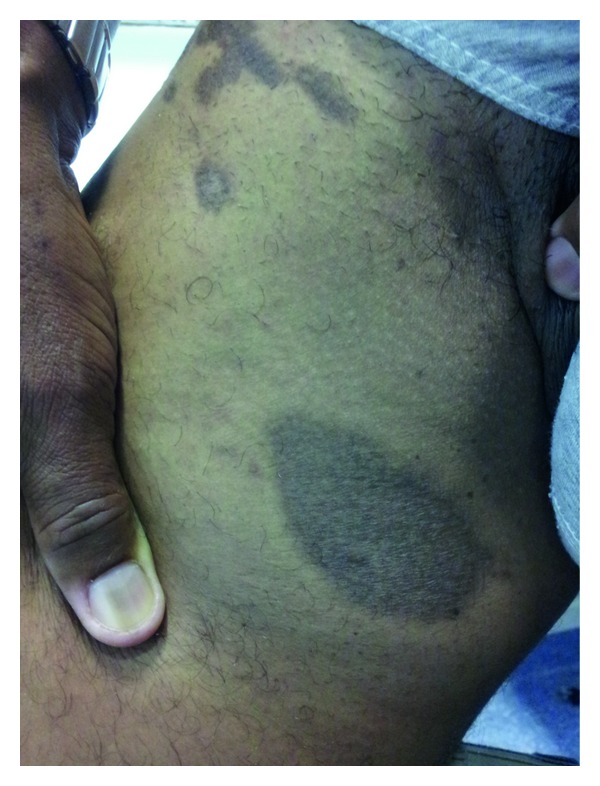
Hyperpigmented plaques of various sizes. Observe the atrophic aspect.

A male patient, 47 years old, natural, and residing in Rio de Janeiro, came to the dermatology clinic due to the appearance of “strange injuries” on his body for the last 6 months. He denies other skin changes and other symptoms, refers to have been diagnosed with Hepatitis C two years ago, and also denies allergies and use of any regular medications on a daily basis. Upon dermatological examination, the following characteristics were observed: hyperpigmented plaques of various sizes and atrophic aspect located in the right inguinal region and thigh (Figure 1).

Biopsy and histopathology confirmed the diagnosis of morphea *en plaque *(late stage), showing thickened collagen bundles infiltrating dermis and subcutaneous fat and atrophic eccrine glands with hypertrophied collagen surrounding them. Laboratory tests showed only the following changes: anti-HCV reactant, altered levels of GOT and GPT, and a positive rheumatoid factor. It is important to mention that the serologies for all other types of hepatitis were negative, as well as for the Scl-70 antibodies and anti-DNA. Serologic screening for *Borrelia burgdorferi *was negative. The patient said that he had refused to perform a liver biopsy at the time of the diagnosis of hepatitis C for the last 2 years.

Given the evidence of morphea *en plaque,* treatment with colchicine cream was established, with further guidance for continued monitoring with a hepatologist and dermatologist. This treatment resulted in stabilization of his cutaneous lesions of morphea.

## 3. Discussion

Through extensive research done on the databases of Pubmed and Cochrane Library, the authors believe that this is the fourth reported case of coexistence and possible association between HCV and LS [5–7]. Clinically, regarding the LS presented by our patient, it is foreseeable that as the disease progresses the skin will become hypopigmented or hyperpigmented in addition to the initially presented atrophy, typical of the scleroderma [8]. It is significant to mention that the diagnosis of LS is based on past history, physical examination, and generally the use of biopsy. There is no confirming laboratory tests or criteria said to be universal for the diagnosis [8]; therefore, this was the procedure adopted towards the patient in question.

At present, the role of HCV in the pathogenesis of autoimmune diseases is not well defined. The replication of the extrahepatic virus cited, especially in mononuclear cells, may cause a suppression mechanism in genetically predisposed individuals to such fact that the process of proliferation of lymphocytes and monocytes is observed in scleroderma [9]. Concomitant to the mentioned, the increase in the function of T-helper cells can result in the stimulation of B-lymphocytes and thereby the synthesis of self-anti-bodies that stimulate the synthesis of collagen [10]. Such factors presented may represent risk factors for the development of scleroderma in patients with HCV.

A recent study performed in the Amazonic region in Brazil supports the association between *Borrelia* and morphea. In this study, immunohistochemical findings suggestive of *Borrelia* infection were observed in 72.7% of the patients. In parallel, spirochetes were detected by focus-floating microscopy in three (50.0%) out of the six suspected cases [2]. Our patient had a negative serology, and he has never been in the Amazonic region, but authors support the hypothesis that geographical aspects can play a role in the association of *B. burgdorferi* and morphea in South America, because cases of this possible correlation have also been reported in Venezuela and Colombia [11].

Clinically, a potential differential diagnosis should be cited: the idiopathic atrophoderma of pasini and pierini (IAPP), since some authors have considered IAPP as a variant of morphea [12–14]. However, our patient do not have the typical cliff-drop border, a slight depression of the skin with an abrupt edge; this represents a peculiar clinical finding in IAPP lesions [15]. The loss of appendageal structures seen in morphea are not a feature of IAPP [15]. 

The use of topic treatment in this event by colchicine is justified, since the LS has an elevated degree of platelet adhesion, and this drug works by regulating the production of type I collagen and adhesion molecules [16]. 

Due to the lack of published cases and the immune mechanism that correlates both diseases, the authors, like Telakis and Nikolaou, recommend in a certain way the research of HCV in patients with LS [6], to offer greater benefits to such research with a possible association.
